# Putative Silicon Transporters and Effect of Temperature Stresses and Silicon Supplementation on Their Expressions and Tissue Silicon Content in Poinsettia

**DOI:** 10.3390/plants9050569

**Published:** 2020-04-29

**Authors:** Jiangtao Hu, Yali Li, Byoung Ryong Jeong

**Affiliations:** 1 Department of Horticulture, Division of Applied Life Science (BK21 Plus Program), Graduate School of Gyeongsang National University, Jinju 52828, Korea; hujiangtao@gnu.ac.kr (J.H.); leeyali@gnu.ac.kr (Y.L.); 2 Institute of Agriculture and Life Science, Gyeongsang National University, Jinju 52828, Korea; 3 Research Institute of Life Science, Gyeongsang National University, Jinju 52828, Korea

**Keywords:** gene expression, Lsi1, Lsi2, silicon content, stress

## Abstract

Silicon (Si) is a beneficial element for plants. To understand Si uptake and accumulation in poinsettia, the Si transporters and their expression patterns were investigated. Nodulin 26-like intrinsic membrane proteins (NIPs) act as transporters of water and small solutes, including silicic acid. In this study, one NIP member, designated EpLsi1, was identified. Additionally, a protein from the citrate transporter family, designated EpLsi2, was identified. Sequence analyses indicated that EpLsi1 belonged to the NIP-I subgroup, which has a low Si uptake capacity. Consistently, the measured tissue Si content in the poinsettia was less than 1.73 ± 0.17 mg·g^−1^ dry weight, which was very low when compared to that in high Si accumulators. The expressions of *EpLsi1* and *EpLsi2* in poinsettia cuttings treated with 0 mg·L^−1^ Si decreased under temperature stresses. A short-term Si supplementation decreased the expressions of both *EpLsi1* and *EpLsi2* in the roots and leaves, while a long-term Si supplementation increased the expression of *EpLsi1* in the leaves, bracts, and cyathia, and increased the expression of *EpLsi2* in the roots and leaves. Tissue Si content increased in the roots of cuttings treated with 75 mg·L^−1^ Si at both 4 and 40 °C, indicating that the transport activities of the EpLsi1 were enhanced under temperature stresses. A long-term Si supplementation increased the tissue Si content in the roots of poinsettia treated with 75 mg·L^−1^ Si. Overall, poinsettia was a low Si accumulator, the expressions of Si transporters were down-regulated, and the tissue Si content increased with temperature stresses and Si supplementation. These results may help the breeding and commercial production of poinsettia.

## 1. Introduction

The effects of silicon (Si) on many flowering plants [1], including poinsettia (*Euphorbia pulcherrima* Willd.) [2], have been investigated, and the results have demonstrated that Si has an effect on plant development. The horticultural traits of sunflowers [3] and gerberas [4] were improved by Si supplementation. Greater biomass, chlorophyll content, and numbers of lateral shoots of *Tagetes patula* were obtained when Si was supplied [5]. However, the characteristics and expression patterns of Si transporters are not extensively studied in these plants. Plants accumulate Si through influx transporters (low silicon 1, Lsi1) and efflux transporters (low silicon 2, Lsi2) [6,7]. Lsi1 belongs to the nodulin 26-like intrinsic membrane protein (NIP) subfamily of the major intrinsic protein (MIP) superfamily [8]. The NIPs have three subgroups named NIP-I, NIP-Ⅱ, and NIP-Ⅲ, which are specific to their substrates [9]. A precise spacing of 108 amino acids (AAs) between the asparagine-proline-alanine (NPA) domains of Lsi1 is essential for Si permeability [10]. A single allelic variation in Lsi1 will result in a different Si uptake [11]. Moreover, suppression of *Lsi1* and *Lsi2* expression results in a reduced Si uptake [6,7]. Therefore, specific protein characteristics and Si transporter expressions are vital to Si uptake by plants.

Studies have shown that Si is beneficial to many plant species, especially when plants are under stresses [12]. It was reported that Si increased the activity of major antioxidant enzymes in plants under high temperature stresses [13]. Si was also observed to increase solutes, membrane stability, and photochemical reactions in plants that are under low temperature stresses [14,15]. Si uptake was inhibited by low temperatures in rice [16] and cucumber [17]. The inhibited transport activities of Lsi2 lead to a reduced Si uptake, while the activities of Lsi1 are not affected by low temperatures [9]. In commercial production, poinsettia propagates in summer and flowers in winter, which exposes it to both high and low temperature stresses. Thus, this study aimed to study how the temperature stresses affect Si transporter expressions and Si accumulation in poinsettia.

In rice [6,7], barley [18], and cucumber [19,20], *Lsi1* and *Lsi2* are expressed mainly in the roots, and their expressions are down-regulated by the Si supplementation. However, the expression of *Lsi1* is not affected by the Si supply in maize [21,22]. Tissue-specific examination of expressions showed that *Lsi1* and *Lsi2* are abundant in the roots and mature leaves, and low in the stems of cucumber [20]. An investigation of Si transporter gene expressions, Si uptake, and Si accumulation has shown that Lsi1 is responsible for the Si transport in the kernel tissues of maize [23]. Moreover, Si distribution and accumulation in the leaves of the grass *Brachiaria brizantha* indicated that mature leaf blades have the highest Si content, followed, in order, by recently expanded leaf blades and non-expanded leaf blades [24].

Overall, although great progress has been made in studying Si transporters and Si accumulation, it is important to explore the expression characteristics of Si transporters and Si accumulation in poinsettia, as the genotype and Si distribution in plant tissues are dramatically different for different species. In this study, Si transporters of poinsettia were analyzed. Furthermore, Si content and expressions of Si transporters in different tissues were determined under high and low temperature stresses and with Si supplementation. The results may help researchers understand the Si uptake capacity better and provide guidance for the genetic breeding of poinsettia.

## 2. Results

### 2.1. Identification of Putative Genes Involved in Si Transport

After assembling and clustering, a total of 111,372 uni-transcripts were obtained. In a TBLASTN search, sequences with significant alignments were retrieved (E-value ≤ 0.01, Table 1). There were 10 putative Si influx and two efflux transporter genes identified in poinsettia. The sequences with the lowest E-values (DN37620_c0_g2_i1 and DN44911_c1_g2_i1) had the longest lengths and matched fragments, and the positives were 64% and 76%, respectively.

### 2.2. Characteristics of the Puatative Si Transporters in Poinsettia

Only one putative Si influx transporter gene and one putative Si efflux transporter gene had complete open reading frames (ORFs) with peptide length of 273 and 523 AAs, respectively. The protein annotation of putative Si influx transporter genes showed that they belonged to the MIP super family, which can selectively transport water, small neutral molecules, and ions. The DN25780_c0_g3_i1, DN25780_c0_g1_i1, and DN49493_c0_g1_i1 had no information in the Pfam database, which might be a result of their short lengths. The putative Si efflux transporter genes belonged to the citrate transporter family, which can translocate and metabolize sodium, arsenate, antimonite, sulfate, and organic anions. The annotation showed that the putative Si influx transporters belonged to the NIP subfamily, and the putative Si efflux transporters had the potential to transport Si (Table 2). As DN37620_c0_g2_i1 and DN44911_c1_g2_i1 had the lowest E-values, when they were searched against the OsLsi1 and SbLsi2, respectively, the longest lengths and matched fragments, and the only complete ORF, they were designated as the *EpLsi1* and *EpLsi2* for the subsequent analyses.

**Figure 1:**
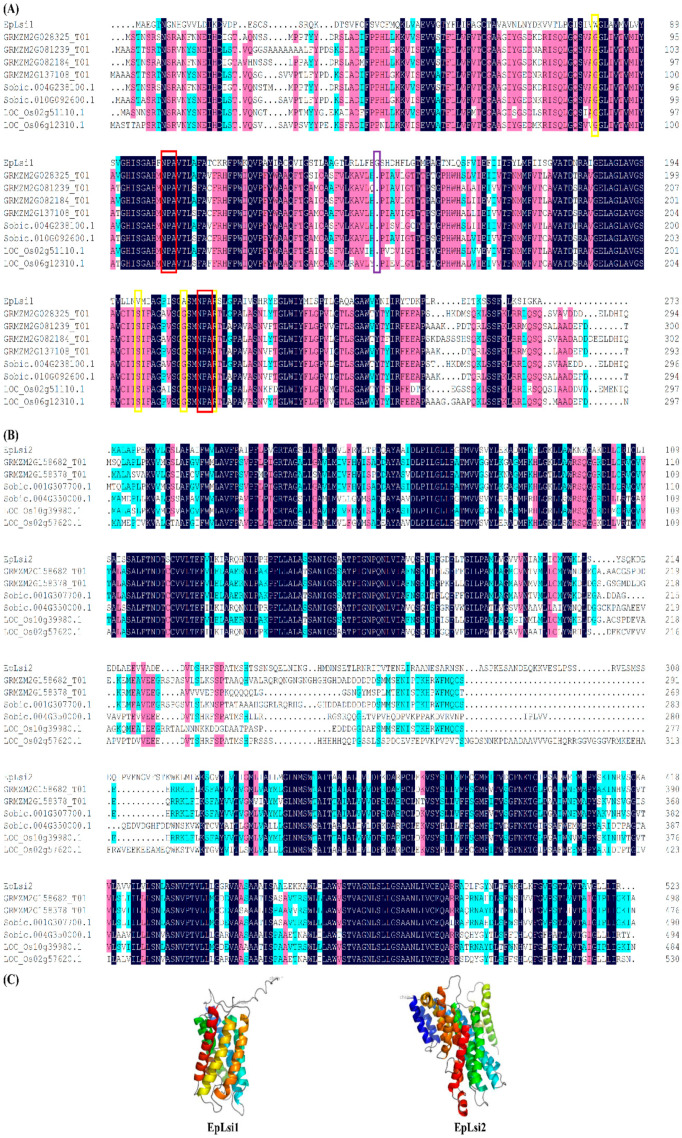
Sequence alignment of the Lsi1 (**A**) and Lsi2 (**B**) amino acids (AAs) in poinsettia, maize, sorghum, and rice; and (**C**) Three-dimensional structure of the EpLsi1 and EpLsi2. The aromatic/arginine (ar/R) selectivity filter, asparagine–proline–alanine (NPA) motifs, and an insertion are boxed in yellow, red, and purple colors, respectively. The putative transmembrane domains are shown in different colors and the remaining loops, coils, and termini are shown in a gray color.

The EpLsi1 contained a tryptophan-valine-alanine-arginine (WVAR) aromatic/arginine (ar/R) selectivity filter, two conserved NPA motifs (Figure 1A), and six putative transmembrane domains (TMDs) (Figure 1C). There were 109 AAs between the NPA motifs with a glycine insertion at the position 142 as compared with the Lsi1 proteins of maize, sorghum, and rice (Figure 1A). Moreover, EpLsi1 shared the highest identity (37%) with GRMZM2G028325_T01, LOC_Os02g51110.1, and LOC_Os06g12310.1. The EpLsi2 shared the highest identity (67%) with LOC_Os02g57620.1 (Figure 1B) and contained 11 TMDs (Figure 1C).

### 2.3. Phylogenetic Analysis of the Putative Si Transporters

**Figure 2:**
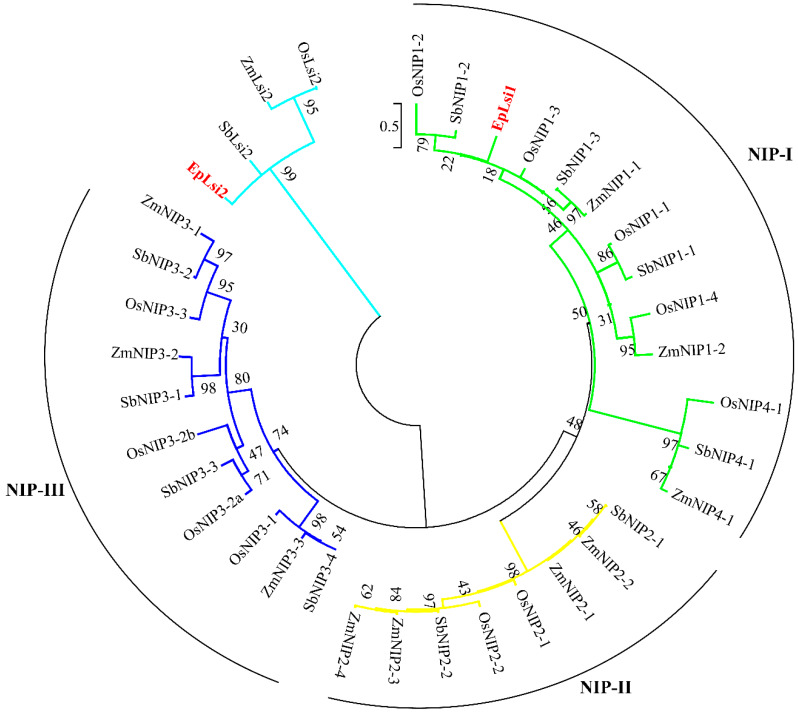
Phylogenetic analysis of the AA sequences of the Si transporters in poinsettia, maize, sorghum, and rice. The sequences used for the construction of the phylogenetic tree are listed in Supplementary File S2.

The putative Si transporters, except EpLsi1 and EpLsi2, were unable to construct a phylogenetic tree as they encoded partial peptides. Based on the phylogenetic tree, it was found that the EpLsi1 was grouped within the NIP-I subgroup (Figure 2).

### 2.4. The Effects of Temperature Stresses and Si Supplementation on the Expressions of EpLsi1 and EpLsi2

**Figure 3:**
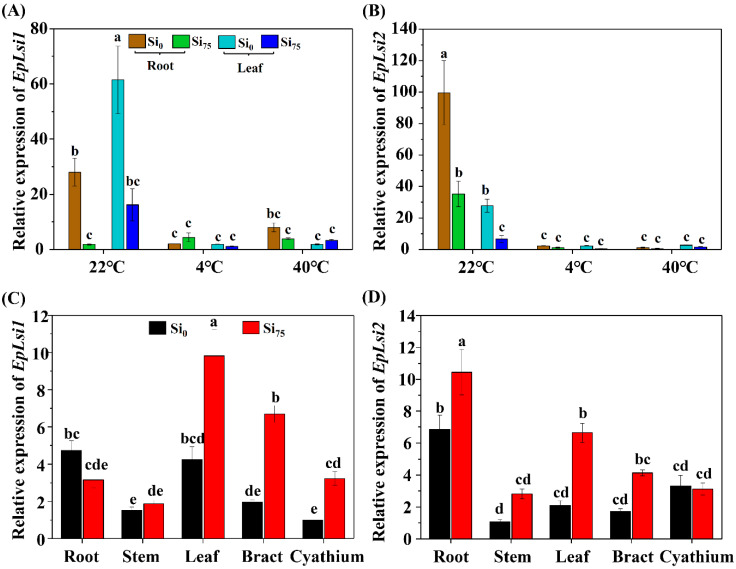
The relative expressions of *EpLsi1* (**A**,**C**) and *EpLsi2* (**B**,**D**) in the roots and leaves of poinsettia cuttings at 22 °C (control), 4 °C (low temperature stress), and 40 °C (high temperature stress) treatments and in the roots, stems, leaves, bracts, and cyathia of poinsettia plants. The data are represented as the mean ± S.E of three biological replicates and three technical replicates. Different letters are significant differences according to Tukey’s test at *p* ≤ 0.05. Si_0_, 0 mg·L^−1^ Si; and Si_75_, 75 mg·L^−1^ Si.

Samples with temperature stress treatments were collected on the fifth day. The relative expressions of *EpLsi1* and *EpLsi2* in the roots and leaves of poinsettia cuttings treated with Si_0_ were down-regulated at 4 °C and at 40 °C (Figure 3A,B). For the poinsettia cuttings treated with Si_75_, the relative *EpLsi1* and *EpLsi2* expressions were not changed at 4 °C and at 40 °C, except for the *EpLsi2* expression in the roots (Figure 3A,B). Short-term supplementation of Si (26 days) significantly reduced the expression levels of both *EpLsi1* and *EpLsi2* in the roots and leaves (*p* ≤ 0.05, poinsettia cuttings under 22 °C), while long-term supplementation of Si (150 days) increased the *EpLsi1* expressions in the leaves, bracts, and cyathia and the *EpLsi2* expressions in the roots and leaves (Figure 3C,D).

### 2.5. The Effects of Temperature Stresses and Si Supplementation on the Tissue Si Content

**Figure 4:**
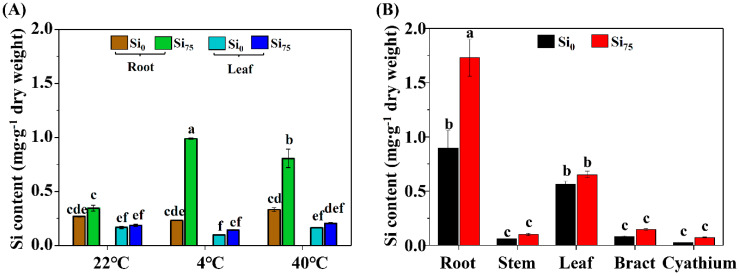
The Si contents in the roots and leaves of poinsettia cuttings at 22 °C (control), 4 °C (low temperature stress), and 40 °C (high temperature stress) treatments (**A**) and in the roots, stems, leaves, bracts, and cyathia of poinsettia plants (**B**). The data are represented as the mean ± S.E of three biological replicates and three technical replicates. Different letters are significant differences according to Tukey’s test at *p* ≤ 0.05. Si_0_, 0 mg·L^−1^ Si; and Si_75_, 75 mg·L^−1^ Si.

The tissue Si content was significantly increased by Si_75_ treatment in the roots of poinsettia under both low and high temperature stresses. However, no significant changes were found in the leaf Si content (*p* ≤ 0.05, Figure 4A). After a long-term Si supplementation, Si_75_ treatment increased the Si content in the roots of poinsettia plants (Figure 4B).

## 3. Discussion

Researchers divide plants into low, moderate, and high Si accumulators based on the ar/R selectivity filter of Lsi1 [25]. EpLsi1 contains a WVAR ar/R filter, which is found in low Si accumulators. The 109 AAs between the NPA motifs might also affect the Si permeability [10]. Moreover, phylogenetic analyses showed that EpLsi1 belongs to NIP-I, while NIP-Ⅲ is reported to transport Si because it possesses a larger pore constriction size [9,26]. The low Si content (less than 1.73 ± 0.17 mg·g^−1^ dry weight) determined in this study confirmed the results of the sequence analyses. Plants categorized as high Si accumulators can accumulate Si at levels of 100 mg·g^−1^ dry weight (10%) or even higher [27]. It is of interest to explore strategies that can increase the Si levels in poinsettia, as Si is often beneficial to plants. Evidence has shown that heterologous expression of the Lsi1 gene increased the Si uptake in rice [19] and *Arabidopsis* [28]. Thus, genetic engineering has the potential to improve Si uptake in poinsettia. In addition to environmental disadvantages, poinsettia also faces problems with poor lateral stem strength [29] and insect attacks [30]. These problems might be alleviated by improving the Si content in poinsettia tissues. A previous study has shown that Si is involved in maintaining the rigidity of rice plants and prevents them from lodging [31]. Moreover, Si may also protect coffee seedlings from insect attacks by constructing a wax layer [32].

How residues affect the Si transport activities of Lsi1 is well understood. However, although studies have proved the Si transport ability of Lsi2 [7,21], the particular residues involved in the Si transport activities have not been identified.

Based on the analyses of Vatansever et al., Si transporters are mainly down-regulated by various stresses including temperature shift [25]. Our results also showed that the relative *EpLsi1* and *EpLsi2* expressions were extremely low under low and high temperature stresses. Moreover, although the Si content was not significantly high in the control group, the relative expressions of *EpLsi1* and *EpLsi2* were dramatically decreased in the roots and leaves of poinsettia cuttings supplemented with Si_75_. Therefore, it can be concluded that the expressions of Si transporters in poinsettia are reduced by low and high temperature stresses as well as by short-term Si supplementation. 

In plants, Lsi1 loads Si into plant root cells, and Lsi2 unloads Si toward and into the xylem [33]. Mitani et al. reported that the Si transport activities of OsLsi1 were unaffected by low temperatures [9]. However, we found that the tissue Si content in the roots of poinsettia plants treated with the Si_75_ increased at both 4 °C and at 40 °C, indicating that the Si transport activities of the EpLsi1 were enhanced under both low and high temperature stresses. Analyses of the tissue-specific Si content showed that Si is mainly accumulated in the roots and leaves of poinsettia. The relative *EpLsi1* expression in the leaves was significantly increased by the Si_75_ treatment. Therefore, it is possible to further increase the Si uptake in poinsettia with foliar sprays of Si solutions. In fact, foliar application of potassium silicate has been reported to reduce bract necrosis in poinsettia [34]. Researchers have also experimented with various Si sources for foliar spray. The effects of different Si compounds were reviewed by Laane et al. [35] It was suggested that stabilized silicic acid is an effective compound for foliar application. Taken together, soil supply and foliar spray may be combined to effectively improve Si uptake in poinsettia.

Notably, expressions of *EpLsi1* and *EpLsi2* are down-regulated by short-term Si supplementation in several plant species [6,7,18,19,20], while the Si content is increased [36]. However, our results showed that Si content was not changed on day 26, which might be a result of the low Si permeability of EpLsi1. Moreover, the expression of the *EpLsi1* increased in the leaves, bracts, and cyathia, and the expression of the *EpLsi2*, increased in the roots and leaves under a long-term Si supplementation. However, tissue Si content only increased in the roots. These results indicate that the movement of Si in the shoot is different from that in the root. In fact, researchers have stated that Si is transported to the lateral organs by transpiration [37,38]. Up to now, no studies have addressed the expressions of Si transporter genes under a long-term Si supplementation. It is interesting to investigate the reasons for the increased expression of *EpLsi2* in the root.

Overall, only one NIP and one Si efflux transporter with a complete ORF were identified in our study, which may contribute to the Si uptake in poinsettia. The deficiency of NIP-Ⅲ leads to low Si uptake. As a result, poinsettia is able to absorb Si, while the accumulation in the tissues is very low. The temperature stresses reduced the expressions of *EpLsi1* and *EpLsi2* in the roots and leaves of poinsettia cuttings treated with Si_0_, but increased the Si content in the roots of poinsettia cuttings treated with Si_75_. Long-term Si supplementation with Si_75_ increased the Si content in the roots, the expression of *EpLsi1* in the leaves, bracts, and cyathia, and the expression of *EpLsi2* in the roots and leaves of poinsettia. Future studies should focus on breeding cultivars with a high Si-accumulation capacity through various techniques.

## 4. Materials and Methods

### 4.1. Plant Materials and Treatments

Terminal cuttings of *Euphorbia pulcherrima* Willd. ‘Flame’ were obtained from mother plants which were grown in the Bas Van Buuren (BVB) medium (Bas Van Buuren Substrates, EN-12580, De Lier, The Netherlands). The cuttings were stuck in a foam wedge medium in plug trays and drenched with a nutrient solution containing 0 (Si_0_) or 75 (Si_75_) mg·L^−1^ of Si supplied from potassium silicate (K_2_SiO_3_). The composition of the nutrient solution was the same as that used in our previous report [2]. After four weeks of culture, the rooted cuttings were subjected to 22 °C (control), 4 °C (low temperature stress), and 40 °C (high temperature stress) for five days in plant growth chambers. Other cuttings were transplanted into 10-cm pots and regularly irrigated with 0 or 75 mg·L^−1^ Si until they flowered.

### 4.2. De novo Assembly and Identification of Putative Si Transporters in Poinsettia

The RNA sequence data of poinsettia in National Center for Biotechnology Information (SRR3509369 and SRR3509535) were *de novo* assembled using Trinity in Galaxy [39] with parameters of 25 kmer word and 300 group pairs distance. The assembled sequences were clustered using the USEARCH (v11.0.667) to obtain the uni-transcript sequences. Then, TBLASTN was performed against AA sequences of Lsi1 and Lsi2 from maize (GRMZM2G028325_T01, GRMZM2G081239_T01, GRMZM2G082184_T01, GRMZM2G137108_T01, GRMZM2G158682_T01, and GRMZM2G158378_T01), sorghum (Sobic.004G238100.1, Sobic.010G092600.1, Sobic.001G307700.1, and Sobic.004G350000.1), and rice (LOC_Os02g51110.1, LOC_Os06g12310.1, LOC_Os10g39980.1, and LOC_Os02g57620.1). The sequences with significant alignments (E-value ≤ 0.01) were retrieved and searched in the Pfam, Swiss-Prot, TrEMBL, and NCBI nr (non-redundant) databases to obtain annotation information.

### 4.3. Sequence Analysis of Si Transporters

The AA sequences were obtained from the Phytozome database [40] and aligned using DNAMAN version 7. The phylogenetic tree was constructed with the maximum likelihood method in MEGA 5.0 and evaluated by 1000 bootstrap replicates. The three-dimensional structure of the proteins was constructed using Phyre2 [41] and visualized with PyMOL v0.99.

### 4.4. Quantitative Real-Time PCR Analysis

Root and leaf samples were collected from the poinsettia cuttings placed under temperature stresses. Root, stem, leaf, bract, and cyathium samples were collected from the potted poinsettia plants. The Easy-Spin Total RNA Extraction Kit (iNtRON Biotechnology, Seoul, Korea) was used to extract the total RNA. After measuring the concentrations, the PrimeScript RT Reagent Kit (Takara, Shiga, Japan) was used to synthesize cDNA. The primers were designed according to the identified sequences (Supplementary Table S1). The reactions were carried out on the Rotor-Gene Q detection system (Qiagen, Hilden, Germany). The relative gene expressions were calculated with the 2^−ΔΔCt^ method. Three biological replicates and three technical replicates were adopted for each treatment.

### 4.5. Determination of the Si Content

The dried root and leaf samples from the poinsettia cuttings under temperature stresses and the root, stem, leaf, bract, and cyathium samples from the potted poinsettia plants were ground into fine powders. One gram of each sample was ashed at 525 °C for 4 hours in a Nabertherm muffle furnace (Model LV 5/11/B180, Lilienthal, Bremen, Germany). The ash was dissolved in 5 mL 25% HCl, and subsequently diluted with 20 mL of warm distilled water. The Si content was then measured three times for each treatment using an inductively coupled plasma (ICP) spectrometer (Optima 4300DV/5300DV, Perkin Elmer, Germany). Each treatment contained three replicates.

### 4.6. Statistical Analysis

The SAS statistical software, Release 8.2, (SAS Inst., Cary. N.C., USA) was used for the statistical analysis, followed by an analysis of variance (ANOVA) and a Tukey′s test (*p* ≤ 0.05).

## Figures and Tables

**Table 1:** Putative silicon (Si) influx and efflux transporter genes identified in de novo assembled data by the TBLASTN.

Gene	Gene ID	E-Value	Length (bp)	Query	Positives ^z^
Si influx transporter	DN37620_c0_g2_i1	6E-066	1006	LOC_Os02g51110.1	149/234 (64%)
	DN37981_c1_g4_i1	1E-043	779	GRMZM2G028325_T01	101/153 (66%)
	DN25780_c0_g2_i1	2E-027	243	LOC_Os02g51110.1	60/78 (77%)
	DN14261_c0_g1_i1	1E-026	302	GRMZM2G081239_T01	64/98 (65%)
	DN37981_c1_g1_i1	4E-017	850	LOC_Os02g51110.1	59/104 (57%)
	DN37620_c0_g2_i2	2E-016	525	LOC_Os02g51110.1	40/56 (71%)
	DN37981_c1_g2_i1	1E-015	479	GRMZM2G028325_T01	46/71 (65%)
	DN25780_c0_g3_i1	5E-010	235	LOC_Os02g51110.1	28/34 (82%)
	DN25780_c0_g1_i1	5E-009	251	Sobic.010G092600.1	33/43 (77%)
	DN49493_c0_g1_i1	1E-004	304	LOC_Os02g51110.1	29/53 (55%)
Si efflux transporter	DN44911_c1_g2_i1	0	1982	Sobic.004G350000.1	402/528 (76%)
	DN11282_c0_g2_i1	4E-034	476	GRMZM2G158682_T01	98/116 (84%)

^z^ Positives were similarities based on the matirx.

**Table 2:** Sequence analyses of the putative genes involved in Si transport.

Gene ID ^z^	Peptide Length (AA)	ORF Integrity	Protein Domain Family ^y^	Annotation ^x^
DN37620_c0_g2_i1	273	Complete	Major intrinsic protein	NIP1-2
DN37981_c1_g4_i1	152	Partial	Major intrinsic protein	NIP5-1
DN25780_c0_g2_i1	80	Partial	Major intrinsic protein	NIP1-2
DN14261_c0_g1_i1	100	Partial	Major intrinsic protein	NIP6-1
DN37981_c1_g1_i1	153	Partial	Major intrinsic protein	NIP5-1
DN37620_c0_g2_i2	81	Partial	Major intrinsic protein	NIP1-2
DN37981_c1_g2_i1	111	Partial	Major intrinsic protein	NIP5-1
DN25780_c0_g3_i1	63	Partial	-	NIP1
DN25780_c0_g1_i1	43	Partial	-	NIP1-2
DN49493_c0_g1_i1	57	Partial	-	NIP2-1
DN44911_c1_g2_i1	523	Complete	Citrate transporter	Si efflux transporter
DN11282_c0_g2_i1	128	Partial	Citrate transporter	Si efflux transporter

^z^ Sequences of the putative Si transporter genes are listed in the Supplementary File S1. ^y^ Protein domain family was searched in the Pfam database. ^x^ Annotation was based on the search results in the Swiss-Prot, TrEMBL, or NCBI nr (non-redundant) databases.

## Supplementary Materials

The following are available online at https://www.mdpi.com/2223-7747/9/5/569/s1, File S1: Sequences of the putative Si transporter genes, File S2: The sequences used for construction of phylogenetic tree, Table S1: Primers for qRT-PCR analysis.
