# Supplementary material for: Putative Silicon Transporters and Effect of Temperature Stresses and Silicon Supplementation on Their Expressions and Tissue Silicon Content in Poinsettia
Source: Plants (Basel). 2020 Apr 29;9(5):569. doi: 10.3390/plants9050569 (PMC7284485; doi:10.3390/plants9050569)
Supplement: Supplementary file 1 [file plants-09-00569-s001.zip › Supplementary Table S1.docx]

**Supplementary Table S1:** Primers for qRT-PCR analysis.

| Gene | Forward (From 5′ to 3′) | Reverse (From 5′ to 3′) | | Tm (℃) |
| --- | --- | --- | --- | --- |
| *EpLsi1* | GGCTCTACGGTTCTCCTG | | CCTGCTTGTGCTCCTAAT | 55 |
| *EpLsi2* | CCGTCCAGTAGGGTAGAGT | | ATGCCAATGGTTACAAGGT | 55 |
| *18s* | ATGATAACTCGACGGATCGC | | CTTGGATGTGGTAGCCGT | 55 |
